# Oxidative stress and starvation in *Dinoroseobacter shibae*: the role of extrachromosomal elements

**DOI:** 10.3389/fmicb.2015.00233

**Published:** 2015-03-25

**Authors:** Maya Soora, Jürgen Tomasch, Hui Wang, Victoria Michael, Jörn Petersen, Bert Engelen, Irene Wagner-Döbler, Heribert Cypionka

**Affiliations:** ^1^Institute for Chemistry and Biology of the Marine Environment (ICBM), Carl-von-Ossietzky University of OldenburgOldenburg, Germany; ^2^Group Microbial Communication, Helmholtz-Centre for Infection ResearchBraunschweig, Germany; ^3^Leibniz Institute DSMZ-German Collection of Microorganisms and Cell CulturesBraunschweig, Germany

**Keywords:** aerobic anoxygenic photosynthesis, starvation, plasmid-maintenance, reactive oxygen species, light-stress adaptation, chlorophyll *a* biosynthesis

## Abstract

Aerobic anoxygenic phototrophic bacteria (AAP) are abundant in the photic zone of the marine environment. *Dinoroseobacter shibae*, a representative of the Roseobacter group, converts light into additional energy that enhances its survival especially under starvation. However, light exposure results in the production of cytotoxic reactive oxygen species in AAPs. Here we investigated the response of *D. shibae* to starvation and oxidative stress, focusing on the role of extrachromosomal elements (ECRs). *D. shibae* possessing five ECRs (three plasmids and two chromids) was starved for 4 weeks either in the dark or under light/dark cycles and the survival was monitored. Transcriptomics showed that on the chromosome genes with a role in oxidative stress response and photosynthesis were differentially expressed during the light period. Most extrachromosomal genes in contrast showed a general loss of transcriptional activity, especially in dark-starved cells. The observed decrease of gene expression was not due to plasmid loss, as all five ECRs were maintained in the cells. Interestingly, the genes on the 72-kb chromid were the least downregulated, and one region with genes of the oxygen stress response and a light-dependent protochlorophyllide reductase of cyanobacterial origin was strongly activated under the light/dark cycle. A Δ72-kb curing mutant lost the ability to survive under starvation in a light/dark cycle demonstrating the essential role of this chromid for adaptation to starvation and oxidative stress. Our data moreover suggest that the other four ECRs of *D. shibae* have no vital function under the investigated conditions and therefore were transcriptionally silenced.

## Introduction

Aerobic anoxygenic photoheterotrophic bacteria (AAP) supplement their energy budget by bacteriochlorophyll *a* (Bchl *a*) based utilization of sunlight (Yurkov and Beatty, 1998). They contribute between 1 and 25% to the marine bacterioplankton abundance (Beja et al., 2002; Oz et al., 2005; Cottrell et al., 2006; Lami et al., 2007; Jiao et al., 2010) and approximately 5% to light-driven electron flow of the surface oceans (Kolber et al., 2000; Jiao et al., 2010). Performing anoxygenic photosynthesis under oxic conditions causes light-induced oxidative stress in AAP. Excited Bchl *a* transfers energy to O_2_ in its triplet state leading to the formation of highly reactive singlet oxygen (Borland et al., 1989) and subsequently to the formation of other toxic reactive oxygen species (ROS) like peroxides and sulfoxides (Glaeser et al., 2011). The response to these toxic by-products has been extensively studied for the anaerobic anoxygenic phototroph *Rhodobacter sphaeroides*. It involves the production of carotenoids as quenchers of ROS (Glaeser and Klug, 2005) and the activation of other detoxifying mechanisms controlled by the alternative sigma factors RpoE (Anthony et al., 2004, 2005), RpoH_I_ (Nuss et al., 2009) and RpoH_II_ (Nuss et al., 2010). These regulators are conserved in AAP and their role in the response to light-induced oxidative stress has been demonstrated for *Roseobacter denitrificans* (Berghoff et al., 2011) and *Dinoroseobacter shibae* (Tomasch et al., 2011), two members of the Roseobacter group abundant in the marine environment (Wagner-Döbler and Biebl, 2006; Brinkhoff et al., 2008).

Under continuous cultivation and carbon limitation *D. shibae* has an increased growth yield in the light (Biebl and Wagner-Döbler, 2006; Tomasch et al., 2011). It is capable of generating ATP by light-driven proton translocation (Holert et al., 2011) and thus employs a photoheterotrophic life style. In our previous study, starving *D. shibae* had a 10-fold increased survival rate as well as higher Bchl *a* and polyhydroxyalkanoate content under optimal light intensity than in the dark. It was also shown that the cells are able to adapt and survive under starvation by sequential changes in cell physiology and gradual changes in morphology (Soora and Cypionka, 2013). Carotenoids serve as quenchers of ROS and thereby prevent photooxidative damages to the cell. Increased carotenoid concentration was observed in *D. shibae* when cells were exposed to high light intensity (Soora and Cypionka, 2013). The light-induced transcriptional changes in *D. shibae* (Tomasch et al., 2011) have recently been proven to be representative for the *Roseobacter* group in the ocean (Ottesen et al., 2014). The expression of genes for pigment synthesis and the photosynthetic apparatus are shut down after light exposure to diminish the effects of ROS (Tomasch et al., 2011). In contrast, the different systems for detoxification of ROS species that include a superoxide dismutase, catalases and glutathione peroxidases were upregulated in response to light. Several of the genes involved in detoxification are located on extrachromosomal replicons (ECRs).

Bacteria of the *Roseobacter* group possess up to a dozen ECRs that in many cases encode ecologically relevant characteristics, e.g., *nir* genes coding for nitrite reduction in *Silicibacter pomeroyi* (Moran et al., 2004) and genes for the production of the antibiotic tropodithietic acid (TDA) in *Phaeobacter inhibens* DSM 17395 (Berger et al., 2012). In *Roseobacter litoralis* and *Sulfitobacter gutiformis, puf* genes coding for subunits of the photosynthesis apparatus are located on plasmids (Pradella et al., 2004). Recently, it was reported that the complete photosynthesis gene cluster (PGC) has been independently translocated from the chromosome to a plasmid in both organisms (Kalhoefer et al., 2011; Petersen et al., 2012). The extrachromosomal elements self-replication and stable maintenance are ensured by their specific replication modules typically containing a replicase and a *parAB* partitioning module (Pradella et al., 2010; Petersen, 2011). These modules also define the compatibility and copy number of individual ECRs, which can vary in size between about 4-kb and several 100 kilo base pairs for cryptic and megaplasmids, respectively. The extrachromosomal elements are either classified according to their compatibility group or to their evolutionary origin, which is reflected by the codon usage (CU) and GC content. Accordingly, ECRs are designated as chromids if their CU is comparable to that of the chromosome or as plasmids if they exhibit a deviant CU (Harrison et al., 2010). *D. shibae* DSM 16493^T^ contains two chromids and three plasmids. The 153-kb and 72-kb chromids have replication modules of the RepA-I and RepB-I type, respectively, whereas the 191-kb, 126-kb and 86-kb plasmids all contain characteristic RepABC-type modules representing the compatibility groups -9, -2 and -1, respectively (Petersen et al., 2013). The two larger 191-kb and 126-kb plasmids exhibit a conspicuous synteny (sister plasmids) and moreover harbor type IV secretion systems (Wagner-Döbler et al., 2009).

During starvation, cells have to save energy and economize the consumption of carbon, nitrogen and sulfur resources. Plasmid replication and maintenance is regulated independently from that of the chromosome and there are several principal options how bacteria can reduce the amount of cellular energy required by ECRs during nutrient limitation. First, gene-expression of particular genes or complete plasmids can be downregulated. Second, the copy number of individual plasmids can be reduced. This option is largely restricted to high- and medium-copy number plasmids. Finally, the cell can cease to replicate non-essential plasmids. Plasmid-digestion is not necessarily disadvantageous for a bacterium, especially if the extrachromosomal element in not required for the survival in its niche and thus alleviates the metabolic burden of DNA replication (Petersen et al., 2013).

Until now, the knowledge about the bacterial gene regulation of ECRs and their role in oxidative stress-response during starvation is limited. In the current study, we investigated the survival strategies of the photoheterotrophic model organism *D. shibae* during starvation. Our analyses based on microarrays and bacterial survival rates prove the beneficial influence of light/dark cycling and dynamic regulation of ECRs on *D. shibae*. Moreover, we could demonstrate the crucial role of a 72-kb chromid for photosynthesis-dependent famine relief.

## Materials and methods

### Cultivation

*Dinoroseobacter shibae* DFL12 (DSM 16493^T^) and two chromid/plasmid-cured mutant strains (Δ72-kb, Δ86-kb; see below) were grown in artificial sea water (ASW) medium with succinate (10 mM) at 25° C, pH 8.0 and 125 rpm. When solid medium was needed, 12 g of agar (Difco) was added per liter. Cultures were starved up to 4 weeks in (a) continuous dark (DD) (Innova 4230, New Brunswick), (b) light-dark cycles (LD, 12 h/12 h, and 12 μmol photons m^−2^ s^−1^, Innova 42, New Brunswick). Starvation experiments were performed as described in Soora and Cypionka (2013). Cells were resuspended in salt solution (ASW without carbon source 100 ml in 250 ml sterile Erlenmeyer flasks) and incubated as described above. Illumination was provided by an 18 watt day light bulb (Osram). Growth was monitored by measuring the optical density (OD) at 650 nm wavelength.

### Cell counting and live/dead staining

Total cell counts were analyzed by SYBR Green staining and epifluorescence microscopy (Olympus BX51). For live/dead staining, 5 μl SYBR Green (fluorescence green) and 7 μl of propidium iodide (PI, fluorescence red) staining solution (10 μg/mL PI in 1 × PBS) were added to the 100 μl cells and stored in the dark for 15 min and viewed under microscope.

### Curing of extrachromosomal elements in *D. shibae*

*D. shibae* DSM 16493^T^ was cured of the 72-kb chromid pDSHI05 (NC_009959.1) and the 86-kb plasmid pDSHI04 (NC_005558.1) with a strategy that was recently described (Ebert et al., 2013; Petersen et al., 2013). The complete RepB-I type replication module of the 72-kb chromid was amplified with P091 (5′-TTGTGAAGAGCCTGAGAAACC-3′) and P092 (5′-TCGATATTGCGGACGGTGAGG-3′). The resulting 4553 bp amplificate was cloned in the pBluescript-SK+ vector containing an additional gentamicin (Gm) resistance cassette and subsequently sequenced in order to validate the absence of PCR errors. Replicon curing of the 72-kb chromid, loss of the Gm curing vector after multiple passaging of the mutants and a PCR-based validation of the presence of the four remaining ECRs was performed as described by Ebert et al. (2013). The 86-kb plasmid of *D. shibae* DSM 16493^T^ was cured in a similar way with the incompatible RepABC-1 type plasmid replication system of *Paracoccus versutus* (Petersen et al., 2009).

### DNA and RNA extraction

DNA was extracted using the AllPrep DNA/RNA Mini Kit (Qiagen) and RNA was isolated using the RNeasy Mini Kit (Qiagen). To avoid RNA degradation, cells were immediately transferred to RNA protect solution (Bacterial RNA protect, Qiagen) and vortexed briefly. The pellets were then frozen at −80°C. For cell lysis, samples were incubated with lysozyme (15 mg/ml, 1 h at room temperature) and then treated according to the manufacturer's instructions. Additionally DNase I treatment using the RNase-free DNase I Set (Qiagen) was performed on the purification column to remove remaining DNA. The purity of the RNA/DNA was controlled by gel electrophoresis and by using a NanoDrop 2000 spectrophotometer (Thermofischer, Germany). The nucleic acids of the LD cultures were extracted after the cells had been illuminated for 2 h. Samples for DNA extraction (2 ml) and RNA extraction (10 ml) were taken separately.

### Microarray experiment and data analysis

Following the procedure described by Tomasch et al. (2011), total RNA was extracted from *D. shibae* cells at the onset of the stationary phase and after starvation (2, 8, and 20 days) under DD and LD conditions. The RNA was labeled using the Universal Linker System (Kreatech) according to the manufacturer's protocol. Data processing and statistical analysis were performed in the R environment (http://cran.r-project.org/) using functions of the LIMMA (linear models for microarray analysis) package (Smyth, 2005) of the BioConductor project (http://www.bioconductor.org/): Background signals were subtracted using the normexp-method. The two channels of one microarray were loess-normalized. Quantile-normalization was applied to make different microarrays comparable. The lmFit and eBayes-functions were used with default parameters to test for differential expression. The obtained *p*-values were adjusted for false-discovery rate using the method developed by Benjamini and Hochberg (1995). Basic R functions were used to assess the Pearson correlation, principal components and hierarchical clustering of the samples based on the normalized probe intensities for the genes. Microarray data have been deposited in the GEO database under accession number GSE62869.

### Design of the plasmid primers

Primers for qPCR/q-RT-PCR (Supplementary Table 1) were designed using Primer-BLAST, which provides the advantage of Primer3 software and BLAST alignment, from NCBI. Targets were the genes coding for the replicases of the respective replicon, i.e., DnaA of the chromosome, RepA-I and RepB-I for the two chromids (pDSHI02, pDSHI05) and RepC-9, RepC-2 as well as RepC-1 for the three plasmids (pDSHI01, pDSHI03, pDSHI04). Primers were purchased from Biomers.net (Germany).

### Standards used for qPCR

For quantification, DNA standards were generated and for this purpose a second primer set (longer amplification product, Supplementary Table 1) was designed for each target gene. These primers were used to amplify of *D. shibae* according to the following protocol: initial denaturation 4 min at 94°C; 36 cycles of 30 sec at 94°C, 45 sec at 59°C (DnaA) or 58.5°C (RepA-I, RepB-I)/59.7°C (RepC-9, RepC-2, RepC-1), and 1 min 72°C; and then a final elongation at 72°C for 10 min. The PCR mixture (50 μl per reaction) contained 5 μl of 10× RedTaq PCR buffer (QIAGEN), 1 mM MgCl_2_, 200 μM dNTPs (Invitrogen, Karlsruhe, Germany), 0.1 pmol/μl (each) primer, 0.05 U/μl of red *Taq* polymerase (QIAGEN), and 5 ng of genomic DNA. PCR products were purified with the PCR purification kit (QIAGEN) and subsequently used as DNA standards for qPCR. The products were analyzed in 1% agarose gels and their concentration was quantified by NanoDrop.

### PCR and qRT-PCR

PCR reactions were performed in duplicates of 50 μl reaction mixture. Template DNA of 2 μl (5 ng/μl) was added to the each reaction mixture containing as described above. *E. coli* DNA was used a control to document that primers are specific to *D. shibae*. For colony PCR, randomly chosen individual colony was added each PCR tube containing the PCR reaction and PCR were performed.

For qPCR, 10 μl of sample was added to 15 μl of a qPCR mixture prepared from SYBR Green PCR Master Mix (Qiagen), which contained each primer at a concentration of 10 pM. The cycle parameters were as follows: 95°C—10 min and 50 cycles of 5 s at 95°C and 15 s at 60. This was followed by 20 s at 82°C. For the determination of the melting curve, the temperature was increased by 1°C every 15 s from 55 to 95°C. For qRT-PCR, 10 μl of RNA samples were added to the 15 μl of a Sybr Green PCR Master Mix but additionally with reverse transcriptase enzyme. The cycle starts with reverse transcriptase parameter: 95°C—10 min and the rest were the same. qPCR and qRT-PCR were performed on a Light Cycler® 480 (Roche).

### Evolutionary analyses

Evolutionary analyses were conducted in MEGA5.2.1 (Tamura et al., 2011). ClustalW was used to align the sequences of putative LPOR proteins. The cyanobacterial LPOR sequences were chosen based on the analysis of Kaschner et al. (2014). The tree was constructed based on the Maximum Likelihood method and a consensus of 1000 bootstrap replicates. NCBI accession numbers of proteins used for alignment and tree construction: YP_001542351.1, WP_025063112.1, WP_026352701.1, KEO90002.1, AGF52872.1, BAC08127.1, BAA25993.2, AAL03934.1, NP_925432.1, YP_001017335.1, YP_730372.1, YP_291269.1, YP_004572862.1, YP_005002484.1.

## Results

### Experimental setup and cell counts during starvation

**Figure 1:**
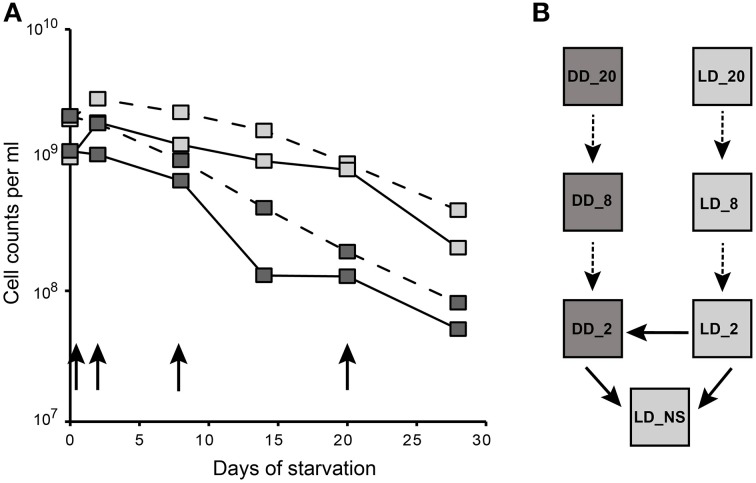
**Experimental design of the starvation experiment. (A)** Total cell counts of *D. shibae* during 4 weeks of starvation and the time points used for microarray analysis. Gray squares represent the light/dark (LD) while the black squares represent the continuous dark (DD) starved samples. The continuous line represents the total cell counts from the current study. Data from our previous study (Soora and Cypionka, 2013) are shown in the dashed lines. The black arrows indicate the sampling points. **(B)** Microarray loop design. Boxes represent samples and are labeled according to their starvation day and condition. The dark and light gray boxes represent the conditions DD and LD respectively. Two color microarrays are represented by arrows connecting the Cy3 and Cy5 labeled samples that are being compared. The continuous arrows mark the microarrays analyzed further.

For the starvation experiment, a culture of *D. shibae*, which was grown in artificial sea water (ASW) medium with succinate under a light/dark cycle until the onset of the stationary-phase, was used as an inoculum (non-starved reference, NS). Cells were harvested, resuspended in artificial sea water medium without substrate and incubated for 4 weeks. During starvation, cells were maintained under dark (DD) and light/dark cycles (LD, 12 h/12 h). Total cell counts determined by SYBR Green staining declined during starvation under both conditions (Figure 1A). Like in our previous study, the decrease in cell numbers was 10-fold higher under continuous dark than under light dark cycles after 4 weeks of starvation (Soora and Cypionka, 2013). Thus, light had a beneficial effect during starvation. The microarray experimental setup comprising an incomplete loop design (Kerr and Churchill, 2007) was designed so that the gene expression could be compared (i) between LD vs. NS as well as DD vs. NS, (ii) between LD vs. DD cells during 2, 8, and 20 days of starvation, respectively (Figure 1B).

### Microarray data quality control

Analysis of the microarray raw signals revealed a decreased reliability of the data with increasing time of starvation. The array signals were fading out for the samples taken after more than 2 days of starvation and showed discordant, low correlation between biological replicates and virtually no genes with acceptable *p*-values (data not shown). This might be explained by a decreased ratio of mRNA to rRNA. Furthermore, the increased number of dead cells in the sample at later time-points might contribute to signal noise. However, an adequate correlation was obtained at day two for both conditions (LD, DD) and for the non-starved reference (NS) (Supplementary Figure 1A). Thus, we focused our comparisons on transcriptomic changes between the non-starved cells and those that were starved for 2 days. A principle component analysis (PCA) was applied to visualize the interrelationships among the samples. The graph shows a clear separation between the NS, LD, and DD samples along the axis representing the first principal component, with the distance between NS and DD being the longest, thus they seem to be the most dissimilar samples (Supplementary Figure 1B). Hierarchical clustering of the samples confirmed the results from the PCA analysis (Supplementary Figure 1C).

### Transcriptome changes at 2 days of starvation

**Figure 2:**
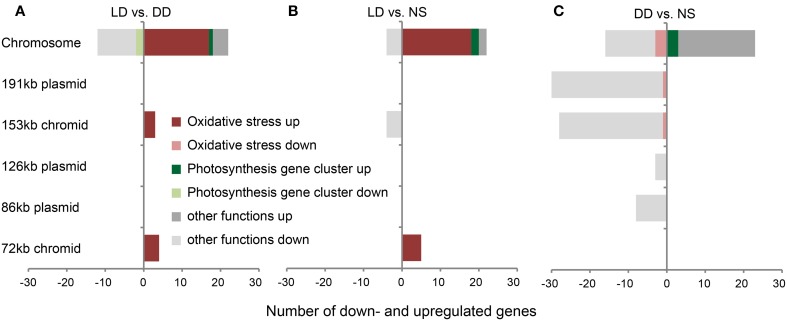
**Substantial changes in gene expression of *D. shibae***. Genes with significant changes in expression on the chromosome and on extrachromosomal replicons (ECRs) of *D. shibae* when **(A)** LD vs. DD, **(B)** LD vs. NS, and **(C)** DD vs. NS are compared. Genes that are part of the photosynthesis gene cluster and the oxidative stress response are highlighted in green and red, respectively.

Out of 4126 protein-coding genes of *D. shibae*, 4088 were represented on the microarray. Using the cut-off criteria of *p* < 0.05 and absolute log_2_-fold changes larger than 1.0, LD vs. DD showed 41 genes, whereas LD vs. NS and DD vs. NS revealed 35 and 108 genes with significant changes, respectively (Figure 2; complete dataset in Supplementary Table 2).

The differential expression of chromosomal genes did not vary substantially between the LD and DD samples. Only 34, 26, and 39 genes showed a significant change in expression for LD vs. DD, LD vs. NS and DD vs. NS, respectively. The comparisons LD vs. DD and LD vs. NS had 18 upregulated genes in common, most of which were part of the stress response of *D. shibae* (Figures 2A,B). Two genes (Dshi_0368, Dshi_0369) are located next to the sigma-/antisigma factor system *rpoE*/*chrR* (Dshi_0366, Dshi_0365). They are controlled by RpoE in *R. sphaeroides* (Anthony et al., 2005) and are part of the light-induced stress response in *D. shibae* (Tomasch et al., 2011). Other genes might also be involved in stress response, for example an AAA+ type ATPase (Dshi_2906), a Clp protease (Dshi_2939) and a cold shock DNA-binding protein (Dshi_0769). Interestingly, the gene encoding the sigma factor RpoH_II_ (Dshi_2978) was strongly upregulated when LD was compared with NS, but downregulated in DD vs. NS. In the photosynthesis gene cluster, *ppaA* a putative regulator of photosynthesis (Dshi_3532) was upregulated under both starvation conditions, whereas the antenna complex genes Dshi_3521 and Dshi_3522 exhibited a decreased expression in both LD vs. DD and LD vs. NS, but were upregulated along with Dshi_3518 under DD vs. NS.

Most of the differentially expressed genes on the ERCs were downregulated, unlike on the chromosome. However, some genes were specifically upregulated: Comparing LD vs. DD, four genes on the 72-kb chromid (Dshi_4167 to Dshi_4170) encoding a transferase, a sulfatase and cytochrome B561 were strongly upregulated with log_2_-fold changes between 1.6 and 2.2. These genes as well as an integral membrane protein (Dshi_4166)—all are part of the oxidative stress response (Tomasch et al., 2011)—also exhibited upregulation comparing LD vs. NS (Figures 2A,B). The remaining three of seven upregulated genes in LD vs. DD, which encode a signal transduction histidine kinase (Dshi_3834), a RNA polymerase sigma factor (Dshi_3835) and a hypothetical protein (Dshi_3836), are all located on the 153-kb chromid and have previously been shown to be part of the stress response, too (Figure 2A). The four-downregulated extrachromosomal genes in LD vs. NS (Figure 2B) are also located on the 153-kb chromid and encode an ATP binding protein of a putative transposase (Dshi_3874), an integrase (Dshi_3875), a transposase (Dshi_3878) and a hypothetical protein (Dshi_3880).

In strong contrast to these findings, a large number of ECR genes displayed downregulation for DD vs. NS (Figure 2C). Interestingly, the genes located on the 72-kb chromid showed no differential expression at all. The 191-kb and 153-kb replicons contained the highest number of downregulated genes (58 of 69), whereas their quantity on the 126-kb and 86-kb plasmids was low. Most of the downregulated genes encoded genetic elements required for genomic reshuffling, like integrases, transposases, and resolvases. Apart from the largest portion of 28 hypothetical proteins, six genes from the 47-kb syntenic region shared between the 191-kb and 126-kb sister plasmids (Wagner-Döbler et al., 2009) were repressed under starvation in the dark.

### Transcriptional silencing of the ECRs

**Figure 3:**
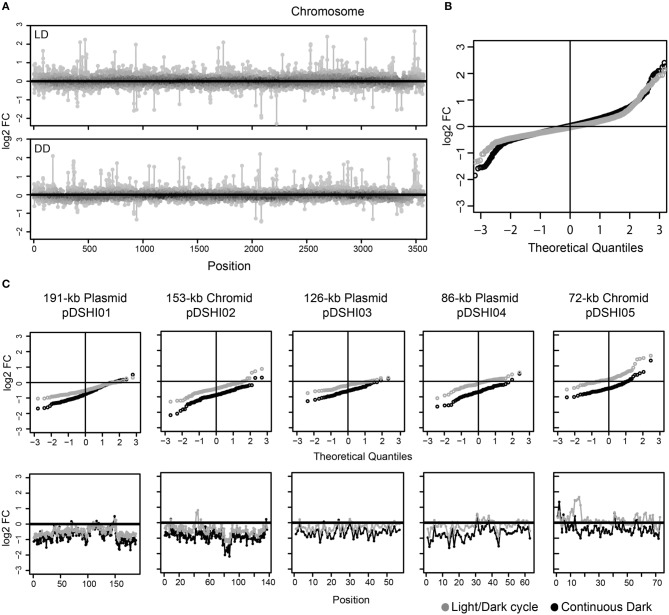
**Overview of differential gene expression of *D. shibae* cells starved for two days. (A)** log_2_-fold changes in chromosomal genes; the upper box represents the light/dark cycle (LD) and the lower portray the dark (DD) starved cells. **(B)** The theoretical quantile-quantile (q–q) plot is shown for both LD (gray) and DD (black) chromosome genes, which are generally used to compare the distribution, showed that they are normally distributed. **(C)** log_2_-fold changes in the ECRs. The upper lane represents the q-q plot and the lower represents the gene expression pattern of the ECRs. Expression under light/dark cycling (LD) and continuous dark (DD) is shown for each gene with gray and black circles, respectively.

The high number of extrachromosomal genes with lower expression under DD compared to NS made us wonder if there was a transcriptional repression of the ECRs or even replicon loss under this condition. Thus, we compared the changes between LD and DD versus NS for all genes separated by replicon, i.e., the chromosome and the five ECRs. Chromosomal genes displayed concurrently up- and down-regulation in both LD and DD conditions as visualized by position specific and quantile-quantile (q-q) plots comparing the actual data to a theoretical normally distributed dataset of the same range (Figures 3A,B). For the ECR genes, the q-q-plots showed that there is indeed a strong shift toward lower expression under starvation in the dark, but, although not as pronounced, also under light/dark cycles in comparison to the non-starved cells (Figure 3C). Thus, transcriptional activity of the ECRs seems to be reduced under both, LD and DD conditions.

**Figure 4:**
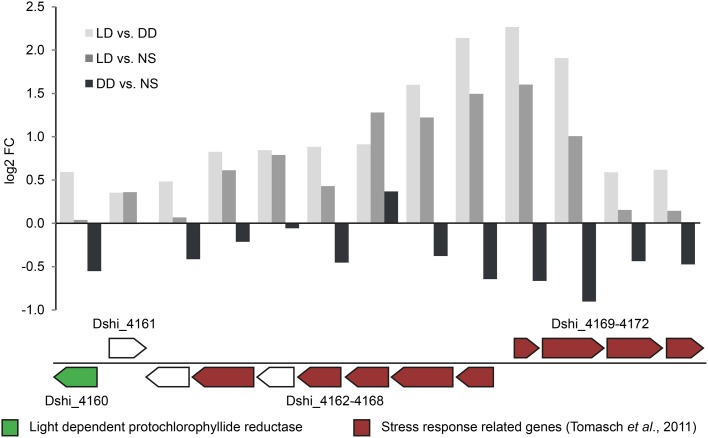
**Expression of the regulated locus on the 72-kb chromid**. The upper bar chart illustrates the differential expression of the regulated locus on the 72-kb chromid (Dshi_4160-4172). In the lower panel, the light dependent chlorophyllide reductase (LPOR) and genes that have been found to be part of the oxidative stress response (Tomasch et al., 2011) are highlighted in green and red colors respectively.

The observed bias was least pronounced for the 72-kb chromid with log_2_-fold changes not exceeding -1 under both conditions. The upregulated genes on this chromid (see above) were located in one particular region (Dshi_4160–Dshi_4172, Figure 4). Interestingly one gene coding for a light-dependent protochlorophyllide reductase (Dshi_4160; EC 1.3.1.33), providing an alternative for catalyzing the second to last step of chlorophyll *a* biosynthesis, is also part of this region that might play an important role during adaptation to light stress under starvation.

In order to corroborate the microarray results, two genes were selected from the 72-kb chromid that were highly induced under the LD condition. Specific primers were designed for the protochlorophyllide reductase and cytochrome B561 (Dshi_4160, Dshi_4149; Supplementary Table 1) as well as for the chromosome-encoded bacteriochlorophyllide reductase (Dshi_3518). The chromosome-encoded DNA gyrase (Dshi_1476) was selected as a reference because it showed a stable expression profile among the different conditions that were compared with each other in the current study. The expression patterns identified for these genes by the microarray and by qRT-PCR showed similar profiles (Table 1). The qRT-PCR results matched the microarray results in most cases except for one contradictory finding (Dshi_3518, LD vs. NS).

**Table 1:** **Comparison of microarray and qPCR expression data from three genes of the 72-kb chromid of *Dinoroseobacter shibae***.

**Locus Tag**	**Scaffold**	**Gene**	**Log_2_ fold changes**					
			**Microarray data**			**qPCR validation (ΔΔC*_t_*)**		
			**LD vs. DD**	**LD vs. NS**	**DD vs. NS**	**LD vs. DD**	**LD vs. NS**	**DD vs. NS**
Dshi_4160	NC_009959	*ldpR*	0.59	0.04	−0.55	8.11	2.02	−6.08
Dshi_4169	NC_009959	*cybB*	2.27	1.60	−0.67	5.24	1.18	−4.06
Dshi_3518	NC_009952	*bchY*	−0.73	1.99	2.72	−2.42	−1.55	0.87

*ldpR, light-dependent protochlorophyllide reductase; cybB, Cytochrome B561; bchY, bacteriochlorophyllide reductase iron protein subunit Y*.

### Quantification of extrachromosomal transcripts

To test the possible loss of ECRs, the abundance and expression of genes that control plasmid replication was assessed by qPCR. The replication initiator DnaA was used to target the chromosome and the replicases specific for each plasmid were used for targeting the ECRs. These genes and their corresponding transcripts (mRNAs) were quantified by qPCR and quantitative Reverse Transcriptase-PCR (qRT-PCR), respectively.

**Figure 5:**
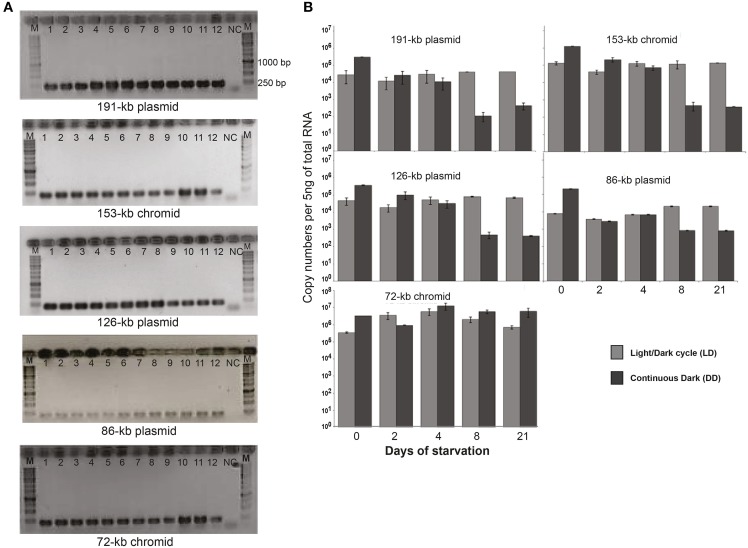
**PCR assay for the detection of the five extrachromosomal elements and the RNA expression profile. (A)** Agarose gels with PCR amplificate of 12 randomly selected colonies of 4 weeks starved *D. shibae* cells. The sizes of the PCR products for ECRs are 201 bp (191-kb), 205 bp (153-kb), 201 bp (126-kb), 208 bp (86-kb) and 201 bp (72-kb). *E. coli* DNA was used as a template for negative control (NC). **(B)** Gene transcript profiles for the five ECRs based on qRT-PCR.

First we tried to quantify the copy numbers of the ECRs and evaluated the plasmid (or chromid) to chromosome (P/C) ratio by qPCR. However, the calculated P/C was in many cases below 0.5 (Supplementary Figure 2). This would mean that not all the bacterial cells had all the plasmids. Accordingly, to investigate whether ECRs are still present in all the cells, *D. shibae* cells were plated on MB agar plates at the last time-point of starvation (4 weeks). Twelve colonies were randomly chosen and colony-PCR was performed with plasmid/chromid-specific primers (only LD condition is shown). The gel electrophoresis showed that all tested colonies still possessed the complete set of ECRs after 4 weeks of starvation (Figure 5A). As we were not able to determine the copy number of the ECRs directly on the DNA level, we measured the expression of the replication genes by qRT-PCR (Figure 5B). Interestingly, at the onset of the stationary phase (day 0), 10-fold increased expression was observed for the replication genes of all ECRs in DD compared to LD cells. After 8 days of starvation the RNA templates declined 1000 fold under DD for all ECRs except the 72-kb chromid while they remained stable in LD. The *repB*-I replication gene of this chromid constantly showed up to 10-fold higher expression compared to the other ECRs under both LD and DD conditions throughout the whole starvation period.

### Survival of replicon-cured mutants under a light/dark cycle

The genes of the 72-kb chromid were the least downregulated of all ECRs under starvation. Furthermore, its replication system was transcribed even under DD after 20 days as shown by RT-qPCR. These results indicate that this chromid might play a special role under starvation, both in the light and in the dark. However, the upregulation of one specific operon (Dshi_4166–Dshi_4170) in the light and the presence of the light-dependent chlorophyllide reductase suggest an important function related to photosynthetic activity. In order to validate our working hypothesis that the 72-kb chromid is specifically important under starvation and light stress, chromid/plasmid cured Δ72-kb and Δ86-kb (control) mutants of *D. shibae* were compared with the wild type strain DSM 16493^T^. All strains were cultivated under the same conditions as before (starvation for 4 weeks) and the optical density as well as the live/dead ratio was measured. Growth of both ECR-cured mutants with 10 mM succinate as carbon source differed remarkably from that of the wild type. Both strains started to grow immediately and reached the maximum OD after 20 h whereas the wild type had a lag phase of 30 h and needed an additional 30 h to reach the maximum OD (Supplementary Figure 3).

**Figure 6:**
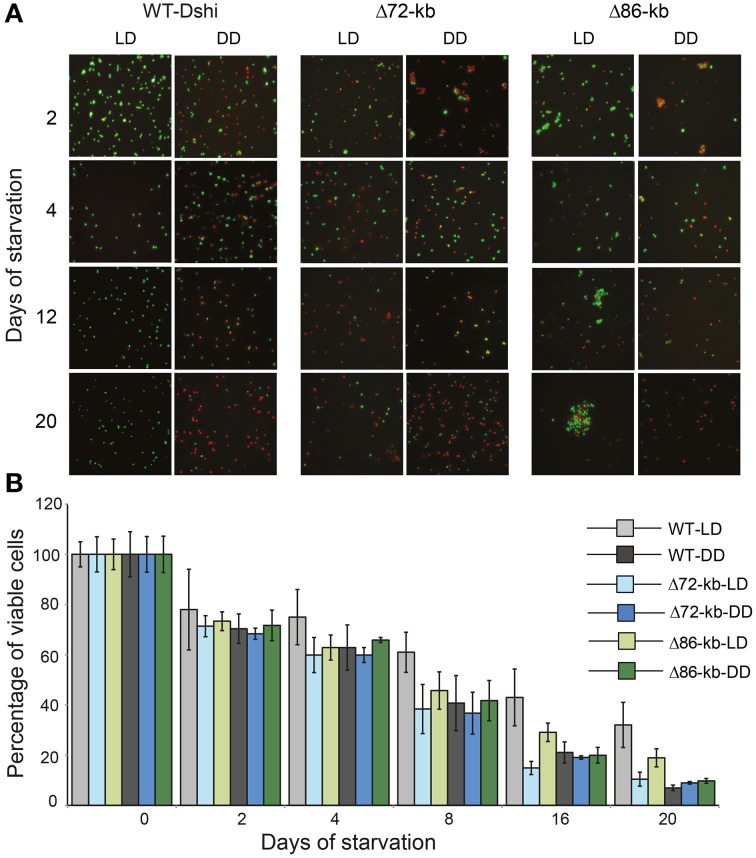
**Microscopic and graphical representation of viable *D. shibae* wild-type and ECR cured cells. (A)** Live/Dead staining where green and red color denotes viable and dead cells, respectively. **(B)** Viability of wild type and plasmid cured mutants (Δ72-kb and Δ86-kb) under LD and DD condition during 3 weeks of starvation.

Live/dead staining and microscopic observation revealed remarkable differences in the ratio of viable (green) to dead cells (red) for those strains depending on the light regimes (Figures 6A,B). After 3 weeks of starvation the wild type *D. shibae* showed a three-fold higher viability under LD compared with DD conditions reaching 32 and 7% of viable cells, respectively. The decrease of living Δ72-kb mutant cells was comparable under both conditions to that of the wild type under DD. After 3 weeks of starvation, only 10 and 9% of viable cells were counted for the LD and DD samples, respectively. The viability of the Δ86-kb mutant decreased slower than that of the Δ72-kb mutant but also reached only 10% of viable cells after 3 weeks in continuous darkness. However, under light/dark cycles 19% of the cells were still viable. Microscopy showed that the Δ86-kb plasmid cured mutant tends to aggregate under LD conditions, a phenotype that has not been observed for the wild type and the Δ72-kb mutant.

## Discussion

In the present study we have shown that the smallest chromid of *Dinoroseobacter shibae* has an essential function during starvation under light/dark cycles. Previously, we had found that *D. shibae* is able to adapt and survive starvation by sequential changes in cell physiology and gradual changes in morphology (Soora and Cypionka, 2013). Here we demonstrate that a major part of this adaptation is dependent on this chromid, while the other four extrachromosomal replicons are silenced.

### Regulation of genes involved in ROS response

Samples from cells that had been starved for 2 days and those from the non-starved inoculum cells passed our quality control and were thus used for further comparisons. Most of the differentially regulated genes on the chromosome were part of the singlet oxygen stress response, as identified by comparison with our previous time-series study of light-dark changes in *D. shibae* (Tomasch et al., 2011). In particular, we found genes belonging to a conserved gene cluster (Dshi_0365–Dshi_0371) that were upregulated in LD cultures when compared to DD cultures and to NS cells. Thus, upregulation appears to be induced by light but more pronounced in starved cells. These results indicate that ROS cause severe problems in *D. shibae* especially under starvation. This conclusion is also supported by our previous study where we reported an increased carotenoid concentration under LD conditions upon starvation which likely functions as a protective response against ROS (Soora and Cypionka, 2013). Recently, a similar high carotenoid-to-Bchl *a* ratio was observed in the *Roseobacter* sp. strain OBYS 0001 under nutrient deprivation (Sato-Takabe et al., 2014).

Only few of the genes in the photosynthesis gene cluster (PGC) showed differential expression. The two genes coding for the light harvesting complex I (Dshi_3521/22) were downregulated in the light but showed the same expression for starved and non-starved cells. The gene coding for the chlorophyllide reductase subunit Y (Dshi_3518; EC 1.3.1.33) was also repressed by light. Although the number of genes in the PGC and the changes in their expression were lower when compared to an actively growing culture (Tomasch et al., 2011), the data are in accordance with another study of a non-dividing *D. shibae* culture under light/dark cycles (Wang et al., 2014). Interestingly, *ppaA* (Dshi_3532), which was shown to code for a regulator of the photosystem in *R. sphaeroides* (Gomelsky et al., 2003), was strongly upregulated in response to light and to starvation. This gene was not affected by light in a growing culture (Tomasch et al., 2011), thus it might play a specific role in the regulation of the PGC under nutrient depletion.

### Silencing of extrachromosomal replicons

The expression of genes on plasmids and chromids was shifted toward lower expression under starvation. We show that this observation was not due to the loss of these replicons. Thus, the ECRs are not degraded as a source of nutrients, like it has been suggested before (Petersen et al., 2013). For *Lactococcus lactis*, it has been shown that several plasmids were lost after 1 year of starvation and regions from two remaining plasmids were rearranged and formed a new ECR (Kim et al., 2001). This indicates that plasmid loss can serve as a long-term strategy for survival under starvation. The same might be true for *D. shibae* and the reduction of copy numbers or systematic silencing of gene expression might be adaptations after shorter times of starvation. Despite the general down regulation of the ECR-located genes after 2 days of starvation we also observed a decreased expression of the replication genes of all ECR except 72kb chromid after 8 days of starvation in the dark, suggesting a second mechanism of copy-number loss or down regulation of the ECRs after this time period.

### Role of the 72-kb chromid

**Figure 7:**
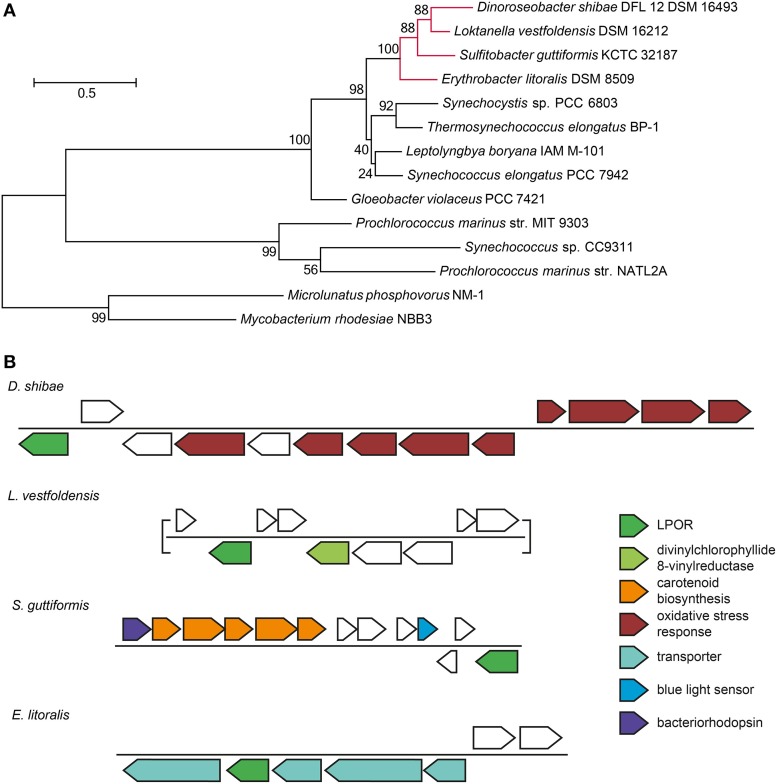
**Light-dependent chlorophyllide reductase (LPOR) in marine *Roseobacter* and *Erythrobacter* strains. (A)** Maximum likelihood tree of the LPOR protein sequences of marine *Cyanobacteria, Roseobacter* and *Erythrobacter* strains. The latter ones are highlighted in red. 1000 bootstrap replicates were performed. The percentage of trees in which the associated taxa clustered together is shown next to the branches. The tree is drawn to scale, with branch lengths measured in the number of substitutions per site. **(B)** Genomic neighborhood of the LPOR gene in *Roseobacter* strains and *Erythrobacter litoralis*. Genes discussed in the manuscript are highlighted.

The expression pattern of the 72-kb chromid was remarkably different from all other ECRs. Under light/dark condition, the bias toward downregulation was the lowest of all ECRs. Furthermore, the expression of the replication genes did not drop at day eight of starvation in the dark. These findings indicate a starvation-specific role for this chromid. The region ranging from Dshi_4160 to Dshi_4171 was even higher expressed in starved cells under light/dark cycles when compared with the positive control. The upregulated genes have previously been identified to play a role in the singlet oxygen (^1^O_2_) stress response in *D. shibae* (Tomasch et al., 2011). One particular gene from this region (Dshi_4160) encodes a light-dependent protochlorophyllide reductase (LPOR, EC 1.3.1.33) of cyanobacterial origin that has recently been proven to be involved in the biosynthesis of Bchl *a* (Kaschner et al., 2014). Among the Proteobacteria, homologous genes are only present in two other roseobacters, i.e., *Sulfitobacter guttiformis* KCTC 32187 (WP_025063112) and *Loktanella vestfoldensis* DSM 16212 (WP_026352701), as well as the *Spingomonadales* strain *Erythrobacter litoralis* DSM 8509 (KEO90002) that has been isolated from a cyanobacterial mat. All four LPOR sequences cluster together apart from the cyanobacterial homologs indicating that they share the same origin (Figure 7A). In *Sulfitobacter* the gene is located next to a region coding for a bacteriorhodopsin, carotenoid-synthesis and a blue light sensor. In *Loktanella vestfoldensis* this gene is located in proximity to another enzyme of the Bchl-*a* synthesis pathway, the divinylchlorophyllide 8-vinylreductase (EC 1.3.1.75). In *Erythrobacter* strain, the LPOR gene is integrated into a locus with genes coding for transporters (Figure 7B). Kaschner and colleagues speculate that this enzyme complements the dark-operative protochlorophyllide reductase (DPOR) encoded in the PGC on the chromosome and thus contributes to adaptation and fine-tuning of Bchl *a* synthesis in fluctuating environments. Their findings support the current results about the specific role of the 72-kb chromid for the adaptation to light/dark cycles.

The complete loss of either the 72-kb or the 86-kb replicon had striking effects, leading to a reduced lag-phase and faster growth in minimal medium with succinate. Thus, the presence of these ECRs seems to be a burden for actively dividing cells. During exponential growth differences between dark and light/dark grown cultures were neither observed for the strain without the 86-kb plasmid nor for the strain lacking the 72-kb chromid. Hence, the genes on the 72-kb replicon do not seem to have an essential role in responding to light-induced stress under these conditions.

The picture completely changes under starvation when the viability of the wild type and the Δ72-kb as well as the Δ86-kb curing mutants is monitored. The Δ72-kb mutant completely lost its ability to benefit from the light energy under the given experimental setup. The viability of the Δ86-kb mutant was also impaired, but this strain still could take advantage from the light. The current study accordingly reflects the specific function of the 72-kb chromid for the adaptation of *D. shibae* to light/dark cycles under starvation. The light-activated genes of this replicon may be involved in an unknown protective mechanism against ROS that is especially important during starvation. It is also possible that the extrachromosomal LPOR (Dshi_4160) is indispensable to keep the photosystems functional when the cells are starved. In contrast to the chromosomal DPOR, no ATP is required for this enzyme to function. Maybe all genes in this region act in concert to support the survival of *D. shibae*. It could also be that the integration site and thus the transcriptional control are important for the acquired LPOR gene to be of advantage for its host as indicated by its localization near genes or gene clusters related to photosynthesis. This hypothesis offers an explanation for the rare occurrence of the LPOR in marine photoheterotrophs: Only few of the random integrations of the horizontally transferred gene result in a transcriptional activity that leads to its positive selection.

## Conclusion

Most bacteria of the *Roseobacter* clade are characterized as ecological generalists with large genomes (Newton et al., 2010) that are often rich in ECRs (Pradella et al., 2010; Petersen, 2011). In contrast to the ubiquitous bacteria with streamlined genomes like *Pelagibacter ubique* (Giovannoni et al., 2005) the wealth of replicons might provide them with the ability to adapt rapidly to changing environmental conditions and various ecological niches (Petersen et al., 2013).

Here we showed that during starvation in *D. shibae*, the activity of the smallest 72-kb replicon with a crucial role for the survival under light/dark cycles is maintained while the other ECRs are silenced. The importance of the 72kb replicon for survival of *D. shibae* in the ocean is already indicated by the fact that it is a chromid, i.e., its codon-usage is similar to that of the chromosome. Thus it has been acquired in the evolutionary past and is now a stable part of the genome. On the one side it seems surprising that such an important trait like light-assisted survival under starvation is dependent on the expression of genes on an ECR. On the other side, the outsourcing of crucial functions from the chromosome to ECRs is one prerequisite for rapid adaptation processes through sharing of larger number of genes by conjugation. Indeed, horizontal gene transfer of photosynthesis genes has frequently been reported. Recent examples include the transfer of the whole proteobacterial PGC to a *Gemmanimonadetes* strain (Zeng et al., 2014) as well as the acquisition of a xanthorhodopsin by a marine *Flavobacterium* (Riedel et al., 2013).

One question yet to be answered is which genes on the 72kb chromid are actually responsible for the beneficiary effects of light under starvation. In particular the role of the acquired light-dependent chlorophyllide reductase has to be unraveled.

### Conflict of interest statement

The authors declare that the research was conducted in the absence of any commercial or financial relationships that could be construed as a potential conflict of interest.
