# Supplementary material for: Oxidative stress and starvation in Dinoroseobacter shibae: the role of extrachromosomal elements
Source: Front Microbiol. 2015 Mar 25;6:233. doi: 10.3389/fmicb.2015.00233 (PMC4373377; doi:10.3389/fmicb.2015.00233)
Supplement: Supplementary file 1 [file DataSheet1.DOC]

**Supplementary Information**

**Oxidative stress and starvation in Dinoroseobacter shibae: The role of extrachromosomal elements**

Maya Soora, Jürgen Tomasch, Hui Wang, Victoria Michael, Jörn Petersen, Bert Engelen, Irene Wagner-Döbler, Heribert Cypionka

1. **Supplementary Figures and Tables**

**1.1 Supplementary Table**

**Supplementary Table 1. Primers for PCR/qPCR used for determination of plasmid copy numbers (PCN) in *D. shibae*.** Chromos, chromosome; Cd, chromid; P, plasmid; *ldpR,* light-dependent protochlorophyllide reductase; *cybB*, Cytochrome B561; *bchY,* bacteriochlorophyllide reductase iron protein subunit Y.

| **Replicon** | **Size (kb)** | **Replicase** | **5´-Primer** | **3´-Primer** |
| --- | --- | --- | --- | --- |
| Chromosome | 3790 | DnaA | TCAGCAACTTTGTGGTCGGA | CTCGGCCGACAAATACAGGA |
| Chromosome^a^ | 3790 | DnaA | CAGAGGTGGCGTTGTACGAT | CCTGAGTGCTGTCCTTACCG |
| pDSHI01 (P) | 191 | RepC-9 | ACAGTCGGCAGAATCCCTTG | GCAGGGATGTCACGTTCCAA |
| pDSHI01^a^ (P) | 191 | RepC-9 | CTAGAACGAGATGCGGACAGG | GACATTCTCATGCCAGGGCT |
| pDSHI02 (Cd) | 153 | RepA-I | GTCCTATGCCCATAACGGGG | ATACGCGTCTCCGGATGTCT |
| pDSHI02^a^ (Cd) | 153 | RepA-I | CGGTATCGTCAGGCGGATTT | AGCCCGACTTCTTCAAGAGC |
| pDSHI03 (P) | 126 | RepC-2 | ACTCTTATGATCTTGAACCTCGCT | GCTGCTCGGAATACGCTTTG |
| pDSHI03^a^ (P) | 126 | RepC-2 | ATTCTCTGATCTTGCGGCCC | ACTCCCCAATTGCTGCCTTT |
| pDSHI04 (P) | 86 | RepC-1 | GTCCGAAGAGATAGCACGGG | AATTGAAAGCTTGCGGCGAA |
| pDSHI04**^a^** (P) | 86 | RepC-1 | CCTCGAACAAGGCGATCTGT | GCTCATCCGTGTCCACATCT |
| pDSHI05 (Cd) | 72 | RepB-I | TATGCCAAGCGGACGG | GAAACAGTTCGACCAC |
| pDSHI05**^a^** (Cd) | 72 | RepB-I | CTATGACACGCGACCC | GCCGCCATCGTATTCA |
| **Locus ID** | **Localization** | **Gene** | **5´-Primer** | **3´-Primer** |
| Dshi_1476^b^ | Chromos | *gyrA* | GGACGGGCAAGGCAACTT | CCATTGACCAGCATGTTCGGA |
| Dshi_4160^b^ | pDSHI05 | *ldpR* | ACGAAATCCCTGATCGACCG | AGATCGTCTGGAACGCCTTG |
| Dshi_4169^b^ | pDSHI05 | *cybB* | CAAGAATGTGGGCGTTCTGC | GTCTCGATCGGGAAGCCC |
| Dshi_3518^b^ | Chromos | *bchY* | GAGATCCTCGACCAG | ACACGAAGGTCAGGCCAT |

a – primers used as a standard for qPCR with product length of 600bp

b – primers used for the validation of microarray results

**1.2 Supplementary Figures**


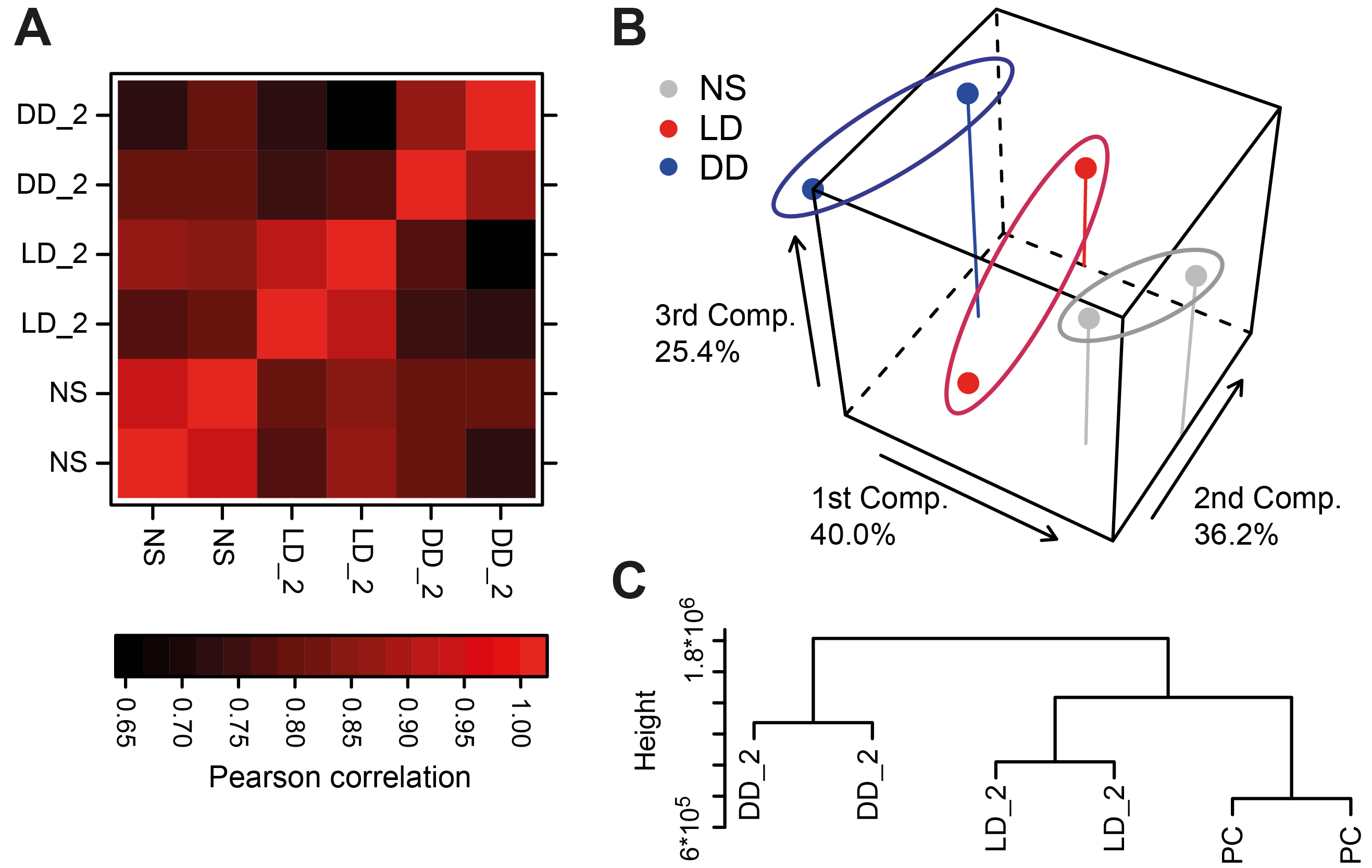


**Supplementary Figure 1.** **Qualitative analysis of microarray dataset by correlation map, principle component analysis (PCA) and hierarchical clustering.** Background-corrected and normalized spot-intensities have been used as input for analysis. (A) Pearson correlation coefficients between gene expression profiles for six samples representing two biological replicates of three cultivation conditions: NS: 0.93, LD: 0.91, DD: 0.86. The color code from black to red indicates the correlation coefficient from low to high, respectively. (B) PCA showing one set of points with non-starved (grey), one with LD (red) and other with DD (blue) samples. The percentage of variance explained by the respective components is shown at the axis. (C) Hierarchical clustering of the samples based on Euclidian distance and Ward’s minimum variance clustering method.

ECR to Chromosome ratio (P/C)

**Supplementary Figure 2.** Extrachromosomal element (ECR) to chromosome (E/C) ratio of *D. shibae* in continuous dark (DD) or light/dark cycles (LD) estimated by qPCR of genomic DNA.


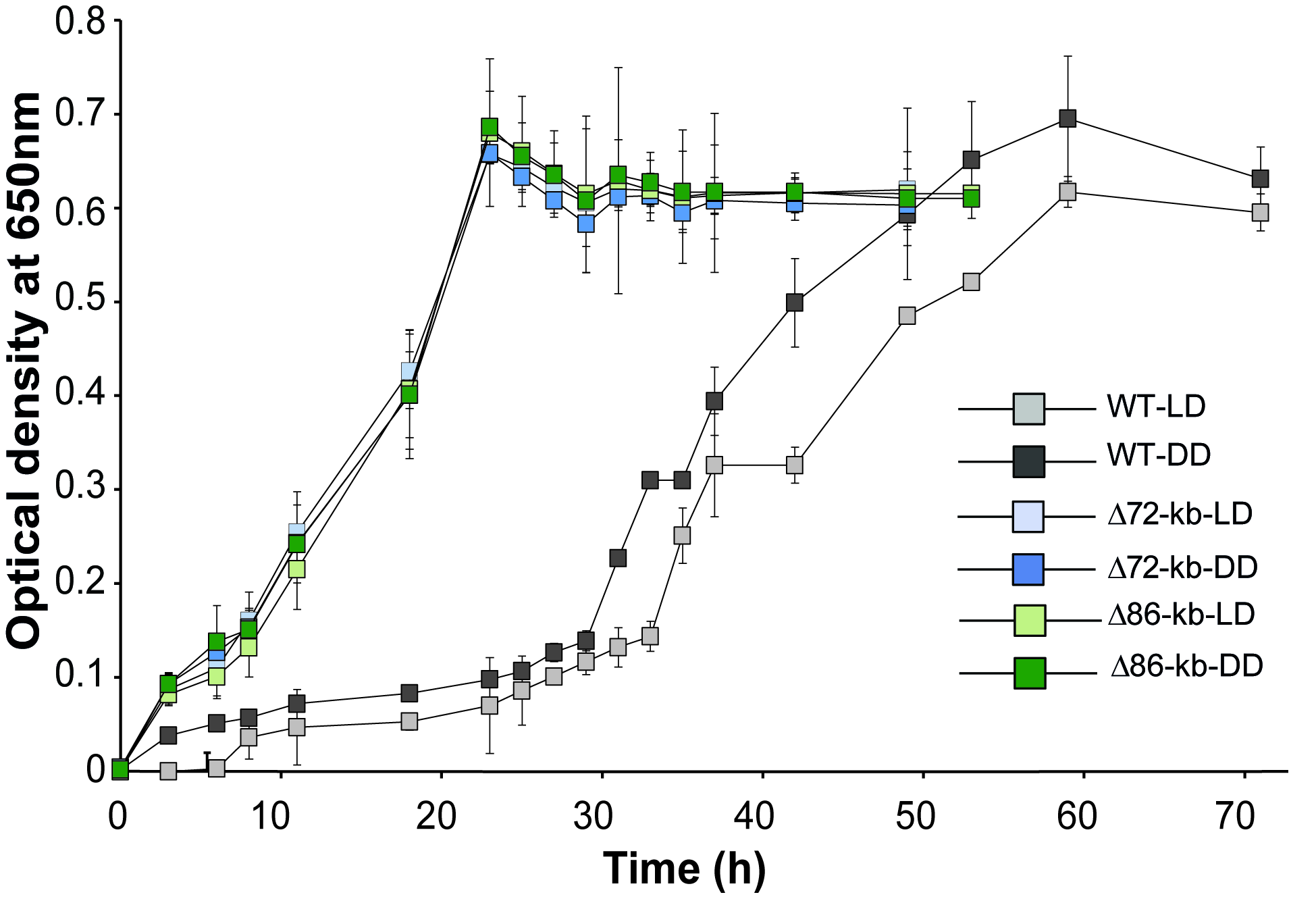


**Supplementary Figure 3.** Growth curves showing the exponential phase of *D. shibae* wild type and Δ72-kb and Δ86-kb replicon cured mutants in continuous dark (DD) or light/dark cycles (LD) with 10 mM succinate as carbon source.
